# How did life become cellular?

**DOI:** 10.1098/rspb.2022.2327

**Published:** 2023-02-08

**Authors:** Aaron D. Goldman

**Affiliations:** ^1^ Department of Biology, Oberlin College, Oberlin, OH, USA; ^2^ Blue Marble Space Institute of Science, Seattle, WA, USA

All organisms on the Earth are composed of one or more cells, making cellularity a fundamental organizing principle of life. Cellular organization as we see it today is complex, requiring the metabolic maintenance of membranes and their constituents as well as genetic control over the timing and coordination of cell division. Despite this complexity, phylogenetic evidence suggests that metabolic pathways for membrane phospholipid biosynthesis had evolved by the time of the last universal common ancestor of life [1]. Some components of a molecular system that embeds proteins into membranes appear to have emerged even earlier than the last universal common ancestor [2], around the same time that the final amino acids were being added to the canonical genetic code. Given the complexity of cellular organization and its reliance on a similarly complex metabolism and genome, the ancient precursors to cellularity must have been significantly simpler.

Prebiotic chemistry experiments suggest that the materials required to make membranes would have been present prior to the origin of life. Specifically, abiotic membranes or protocells can form from naturally occurring or otherwise prebiotically plausible lipids [3]. Protocells are also favoured to form under conditions that are similar to settings in which the origin of life may have taken place [4], specifically alkaline hydrothermal vents in the early ocean. It is possible, therefore, that protocells were present throughout the earliest evolutionary history of life. The potential role of these precursor protocells in the origin of life and their subsequent evolution into complex cellular organisms remain crucial topics in the study of very early evolutionary history.

The RNA World hypothesis proposes that the current tripartite genetic system composed of DNA, RNA and proteins was preceded by a stage in which functional RNAs including RNA enzymes, or ribozymes, were encoded by RNA genes [5]. Whether or not this hypothesis is correct, recent laboratory experiments have demonstrated several synergistic relationships between abiotic membranes and nucleobases or nucleic acids. For example, nucleobases have been shown to stabilize membranes composed of the prebiotically plausible compound decanoic acid [6]. At the same time, protocell vesicles appear to enhance the rate of RNA replication by polymerase ribozymes and also increase the rate of evolutionary adaptation [7]. Thus, the nucleobase constituents of ribozymes can stabilize protocells, while the protocells themselves can enhance both the function and evolution of ribozymes.

A parallel line of theoretical and computational research suggests that natural selection would have favoured cells or protocells during the early evolution of life because of their ability to stabilize genomes. For example, if the genetic material of early life forms was encoded on small, single-gene chromosomes rather than larger multigene chromosomes, these gene-sized chromosomes may have replicated at different rates, disturbing the balance of gene products. Protocell encapsulation could have mitigated inter-gene conflict and ensured that at least some progeny received the correct complement of genes after protocell division [8]. Recent computational simulations have shown that encapsulation within protocells could have also reduced the negative effects of parasitic genes on early life forms and allowed the genetic system to accommodate higher mutation rates [9]. Other computational simulations showed a coevolution between cellularity and metabolism where selection favouring less permeable cell membranes was attributed to the cell's ability to limit random horizontal gene transfers and thus maintain its suite of metabolic genes [10].

Taken together, these and related lines of research show that different selection pressures favouring genome integrity also promote the evolution of protocell encapsulation or cellular organization. These simulations, however, all assume that some kind of heritable genes, such as those imagined by the RNA World hypothesis, already exist, and that evolution is acting on those genes as units of selection. A recent publication by Nunes Palmeira *et al*. [11] is unique among computational protocell simulations because it is devoid of genes and is based entirely on chemical flux through a protometabolic network. As a result, the model can investigate potential protocell dynamics prior to the evolution of genetic control and natural selection.

In their model, CO_2_ can enter a protocell wherein it is fixed to form the two-carbon compound acetate, a process that is plausible in certain potential origin of life settings, namely iron sulfur minerals and alkaline hydrothermal vent systems. The fixed carbon can then be converted through various protometabolic pathways into prebiotic amino acids, sugars or fatty acids, or it can be consumed in order to store chemical potential energy in the form of phosphorylated acetate. As fatty acids accumulate, these are incorporated into membranes, which leads to protocell growth and division. One subset of the amino acids in this protometabolic network can facilitate the formation of iron–sulfur clusters, an important enzyme cofactor that catalyses redox reactions across a range of metabolic pathways. The other subset of amino acids may be combined with sugar and energy to generate nucleotides.

An important feature of this model is that nucleotides are treated as catalysts rather than components of a genetic polymer such as RNA. Many reactions in modern metabolism, especially thermodynamically unfavourable reactions, are catalysed by cofactors that are composed of nucleotides or nucleotide derivatives. ATP, for example, is both the adenosine nucleotide used in RNA synthesis and the universal currency of chemical potential energy used throughout metabolism by every organism. NADH, which is similarly used throughout metabolism as a carrier of redox potential energy, is a dinucleotide composed of an adenosine nucleotide and a nicotinamide nucleotide. Other examples of nucleotide- or nucleoside-based cofactors include NADPH, FADH_2_, FMN, coenzyme A, *S*-adenosyl methionine and UDP-glucose.

The prevalence of nucleotide cofactors in metabolism was described several decades ago as evidence for an RNA World, the idea being that nucleotide cofactors are remnants of ribozyme active sites in which the surrounding scaffold has since been replaced by protein enzymes [12]. Nunes Palmeira and colleagues reverse this chronology, suggesting that individual nucleotides could have acted as prebiotic catalysts promoting protometabolism prior to the emergence of ribozymes or any other genetically encoded enzyme. The authors model several different scenarios in which nucleotides can catalyse different branches of the protometabolic network. In doing so, they discover that nucleotide catalysis can facilitate protocell growth and division in some scenarios, but not in others.

When nucleotides predominantly catalyse fatty acid synthesis, this has a counterintuitive long-term effect of undermining protocell growth and division. Though fatty acid synthesis should lead directly to protocell growth and division, diverting the metabolic flux toward fatty acid synthesis also decreases the rates of synthesis of other products that would otherwise feed back on the protometabolic network. Specifically, fatty acids represent an endpoint in the network and thus do not contribute to the synthesis of nucleotides from amino acids, sugars and energy. They also do not contribute to increased carbon fixation, i.e. the input into the protometabolic network. Therefore, when metabolic flux is diverted to fatty acid synthesis, the rates of metabolism ultimately slow down overall and lead to decreased, rather than increased, protocell growth and division.

Alternatively, protocell growth and division is enhanced when nucleotide catalysis creates a positive feedback loop within the protometabolic network. This is best achieved when nucleotides catalyse carbon fixation, thereby increasing the input of reactants into the entire protometabolic network. Protocell growth and division can also be increased through nucleotide catalysis of amino acids and energy currency. In both of these cases, the increased production of amino acids or energy currency has the ultimate effect of increasing carbon fixation. So even though, in this scenario, the nucleotides catalyse downstream pathways in the protometabolic network, this results in a subsequent acceleration of input into the entire network.

The authors find that the protometabolic network is most successful when nucleotide catalysis is general and flux remains balanced through all branches of the network. This result is especially satisfying because the sort of general catalysis that promotes protocell growth and division in the model is similar to the general catalysis that we see performed by the nucleotide cofactors that drive metabolism today. Nucleotide cofactors like ATP and NAD are used by many enzymes across many different metabolic pathways. As in the protometabolic network model, these cofactors in modern metabolism are essential in carbon fixation as well as the downstream synthesis of biomolecules. As Nunes Palmeira and colleagues point out, nucleotide cofactors even catalyse their own biosynthesis; that is, ATP (a purine nucleotide) is an essential component of purine biosynthesis pathways.

This protometabolic network, though theoretical, is also supported by recent research in prebiotic chemistry. For example, a recently discovered abiotic reaction network synthesizes amino acids and other metabolites from small organic compounds that themselves can be synthesized through abiotic carbon fixation [13]. Despite recent progress, however, a complete protometabolic network such as that described by Nunes Palmeira and colleagues seems far from achievable at present. To that end, however, their model offers both a blueprint for future experimental research in prebiotic protometabolism and an important theoretical bridge between prebiotic chemistry and the kinds of properties that we associate with life beyond its chemical composition, specifically cellular organization and genetic inheritance.

As the authors point out, in a protometabolic network catalysed by nucleotides, random polymers of RNA or peptides may be able to bind and stabilize nucleotide cofactors, enhancing their catalytic functions. Protocells that incorporate these kinds of polymers may grow and divide faster as rates of carbon fixation and general flux increase through the protometabolic network. Experimental evidence supports such a scenario, demonstrating that non-enzymatic, template-directed copying of RNA can occur within protocells [14] and that the presence of a beneficial catalyst within protocells causes those protocells to outcompete others [15]. Thus, the first genes could have emerged as a consequence of protocell competition via a nucleotide-catalysed protometabolic network.

The model described by Nunes Palmeira and colleagues thus depicts a first step in establishing the deep relationship between genetic inheritance and cellular organization in early evolutionary history. Protocells were previously shown to have been stabilized by nucleobases [6]. Here, they are growing and dividing owing to the presence of catalytic nucleotides [11]. Nucleotide-driven catalysis could have been further enhanced by the polymerization of nucleotides into short nucleic acids, and the protocells that contained them would have outcompeted protocells that did not [14,15]. As the first genetic polymers emerged from such a system, protocells would have increased the catalytic efficiency of those polymers and promoted faster evolutionary adaptation [7]. Ultimately, as these templates evolved into genes and genomes, selection pressure may have promoted the transition from protocell encapsulation to *bona fide* cellular organization in order to stabilize those genomes [8–10]. This synergistic relationship between the origin of genes and the origin of cellularity may explain why cellular organization appears to have emerged very early in the history of life on Earth and why it has not been lost during the billions of years of evolution that followed.

## Data Availability

This article has no additional data.
